# Bibliometric analysis of colchicine in cardiovascular health: trends, key contributors, and global collaborations

**DOI:** 10.55730/1300-0144.6003

**Published:** 2025-03-26

**Authors:** Meltem SERTBAŞ, Özlem ÖZAYDIN, Habip YILMAZ, Abdullah Emre GÜNER, Özden GÜDÜK, Nalan OKUROĞLU, Ali ÖZDEMİR, Yaşar SERTBAŞ

**Affiliations:** 1 Department of Internal Medicine, Fatih Sultan Mehmet Training and Research Hospital, Health Sciences University, İstanbul, Turkiye; 2 Türkiye Institute of Health Policies, TÜSEB, İstanbul, Turkiye; 3 Department of Anesthesia and Reanimation, Sultan Abdulhamid Training and Research Hospital, Health Sciences University, İstanbul, Turkiye; 4 Department of Public Health, Hamidiye Medical Faculty, Health Sciences University, İstanbul, Turkiye; 5 Department of Healthcare Management, Faculty of Economics, Administrative and Social Sciences, Istinye University, İstanbul, Turkiye; 6 Department of Internal Medicine, Fatih Sultan Mehmet Training and Research Hospital, Health Sciences University, İstanbul, Turkiye; 7 Department of Internal Medicine, Fatih Sultan Mehmet Training and Research Hospital, Health Sciences University, İstanbul, Turkiye; 8 Department of Internal Medicine, Fatih Sultan Mehmet Training and Research Hospital, Health Sciences University, İstanbul, Turkiye

**Keywords:** Colchicine, cardiovascular health, bibliometric analysis

## Abstract

**Background/aim:**

The study aims to systematically analyze the body of literature concerning the effects of colchicine on the cardiovascular system, providing a comprehensive evaluation of the current knowledge and highlighting the clinical and scientific relevance of colchicine in this field.

**Materials and methods:**

Data were obtained from the Web of Science (WOS), Scopus, and PubMed databases on March 17, 2025. The search terms included “Colchicine” AND (“Cardiovascular Diseases” OR “Heart Diseases” OR “Coronary Artery Disease” OR “Myocarditis” OR “Pericarditis” OR “Atherosclerosis” OR “Heart Failure” OR “Myocardial Infarction” OR “Ischemic Heart Disease” OR “Acute Coronary Syndrome” OR “Cardiac Arrhythmias” OR “Thrombosis” OR “Stroke”). No restrictions were applied regarding publication date or language, ensuring the comprehensive retrieval of relevant data. Publications were categorized according to their document type and indexes. Additionally, a cooccurrence analysis of key words was conducted using VOSviewer 1.6.18.

**Results:**

A total of 425 publications on the effects of colchicine on the cardiovascular system were identified, published between 1976 and 2025. There has been a significant increase in research since 2015. Most publications were in the Cardiac Cardiovascular Systems category. “Colchicine” was the most frequently used key word, appearing 170 times. The USA ranked first in publication count, with 163 studies. The USA, Italy, Canada, and China played significant roles in global research collaboration.

**Conclusion:**

This bibliometric analysis demonstrates the increasing importance of colchicine as a therapeutic agent in the management of cardiovascular disease.

## 1. Introduction

Colchicine is an alkaloid that is naturally derived from the plant *Colchicum autumnale*. It has been used since ancient times for the treatment of joint pain and is particularly effective in treating gout, familial Mediterranean fever (FMF), and pericarditis due to its potent antiinflammatory properties [1,2]. The active compound in the plant, colchicine, is notable for its regulatory effects on inflammatory processes.

The effect of colchicine is attributed to its capacity to inhibit various mechanisms of inflammation at the cellular level. One of the most critical mechanisms of action is its ability to suppress inflammatory cell function through the inhibition of tubulin polymerization and microtubule formation. This action effectively prevents the migration of neutrophils to the site of inflammation, thereby diminishing the severity of the inflammatory response.

Furthermore, colchicine inhibits the release of proinflammatory cytokines, such as interleukin-1β (IL-1β), by blocking the activation of NLRP3 inflammasomes, which are essential mediators of inflammation. Consequently, colchicine is considered to reduce the pathophysiological processes that contribute to the progression of cardiovascular diseases through its antiinflammatory effects [3–5].

With the idea that colchicine’s antiinflammatory effects may prevent the development of diseases in the cardiovascular system, various studies on this topic have been conducted since the early 1980s. Furthermore, there has been a noticeable increase in interest in this field in recent years, resulting in a growing number of research activities.

Given that atherosclerosis is fundamentally driven by inflammatory processes, numerous clinical studies and reviews have consistently reported favorable outcomes supporting the hypothesis that colchicine may lower the risk of cardiovascular events by mitigating inflammation.

Among the existing studies, the most prominent are the LoDoCo and COLCOT trials, both of which demonstrated that colchicine treatment reduces the incidence of cardiovascular events in patients with stable coronary artery disease and those who have experienced myocardial infarction [6,7]. However, there remains a gap in comprehensive studies that systematically examine the existing literature on colchicine—particularly in identifying influential publications, tracking research trends, analyzing publication sources, and mapping collaboration networks.

This bibliometric study aims to systematically analyze the body of literature concerning the effects of colchicine on the cardiovascular system, providing a comprehensive evaluation of the current knowledge and highlighting the clinical and scientific relevance of colchicine in this field.

## 2. Methods

This retrospective bibliometric analysis aimed to evaluate the scientific literature on the cardiovascular effects of colchicine. To ensure comprehensive coverage, a systematic search strategy was developed using a combination of Medical Subject Headings (MeSH) terms and relevant key words. The final search query included: “Colchicine” AND (“Cardiovascular Diseases” OR “Heart Diseases” OR “Coronary Artery Disease” OR “Myocarditis” OR “Pericarditis” OR “Atherosclerosis” OR “Heart Failure” OR “Myocardial Infarction” OR “Ischemic Heart Disease” OR “Acute Coronary Syndrome” OR “Cardiac Arrhythmias” OR “Thrombosis” OR “Stroke”). The search was conducted within the title fields of three major databases—Web of Science (WOS), Scopus, and PubMed—to ensure the retrieval of highly relevant publications.

The data collection process was carried out on March 17, 2025. In the initial stage of the search, records were retrieved from three databases: Web of Science (n = 454), Scopus (n = 450), and PubMed (n = 362). The inclusion criteria were defined as follows: original research articles, review papers, editorials, meeting abstracts, and proceeding papers that focused on the cardiovascular effects of colchicine. Based on these criteria, the number of relevant publications was refined to 395 in Web of Science, 381 in Scopus, and 208 in PubMed. No restrictions were applied regarding publication date or language, ensuring comprehensive data collection. Subsequently, the datasets were merged, and duplicate entries were removed, resulting in a final dataset comprising 425 unique publications for bibliometric analysis.

To enhance reproducibility and transparency, a structured step-by-step methodology was implemented for data retrieval and analysis. Bibliographic data from the selected publications were extracted and systematically processed to assess key bibliometric indicators, including:

- Annual publication trends
- Citation counts and distribution
- Authorship and institutional productivity
- Country-level contributions and collaboration networks
- Key word frequency and cooccurrence patterns
- Source/journal analysis
- Funding agency contributions

These parameters were analyzed using VOSviewer (version 1.6.18) to identify major research themes, conceptual structures, and evolving trends within the selected body of literature.

This study does not require ethical approval, as it is a retrospective analysis based on publicly available data. Additionally, informed consent was not required due to the retrospective nature of the study.

## 3. Results

A total of 425 publications addressing the cardiovascular effects of colchicine were identified, spanning the period between 1976 and 2025. Upon classification, the dataset included 200 original research articles, 107 meeting abstracts, 66 review papers, 51 editorial materials, and 1 proceeding paper.

When the studies were analyzed based on their indexing status, it was observed that 391 publications were indexed in the Science Citation Index Expanded (SCI-EXPANDED), 35 in the Emerging Sources Citation Index (ESCI), 77 in the Conference Proceedings Citation Index, and 1 in the Social Sciences Citation Index (SSCI).

**Figure 1:**
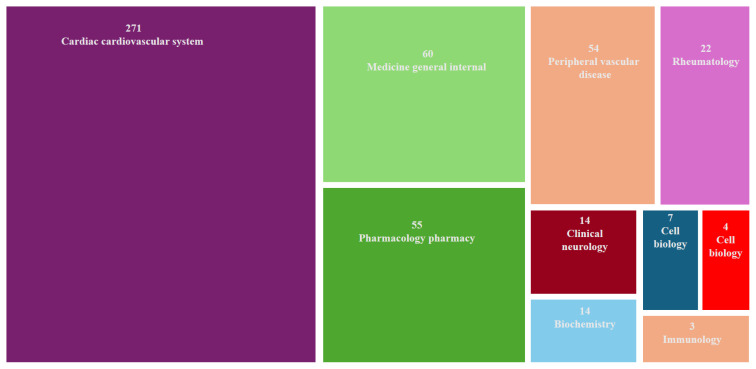
Distribution of publications on colchicine’s effects on the cardiovascular system across various scientific disciplines.

As shown in the tree map chart created for a total of 425 publications, the studies were categorized into various scientific disciplines. The most frequently observed category was “Cardiac & Cardiovascular Systems”, which included 271 records (61.7%). This was followed by “General & Internal Medicine” with 60 records (14.1%), and “Pharmacology & Pharmacy” with 55 records (12.9%). Other subject areas included “Peripheral Vascular Disease” with 54 records (12.7%), and “Rheumatology” with 22 records (5.1%), along with several others with lower representation. This distribution of subject categories highlighted the significant relevance of colchicine in cardiovascular research (Figure 1).

**Figure 2:**
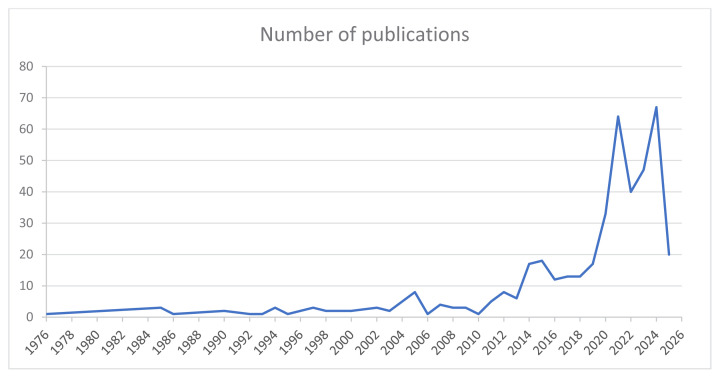
Number of publications by year.

As shown in Figure 2, publication activity was modest until 2015 but then increased sharply in the following years. Peaks were observed in 2021 and 2024, with 64 and 67 publications, respectively. Despite covering only the first quarter, 2025 already showed 20 publications, underscoring the sustained research interest in colchicine’s cardiovascular effects.

**Figure 3:**
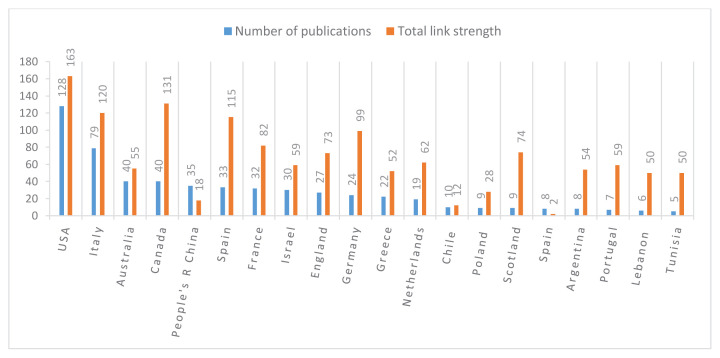
Number of publications and collaborative link strength across countries in cardiovascular research on colchicine.

As shown in Figure 3, the United States ranked first with 128 publications, followed by Italy (79), Australia (40), Canada (40), and the People’s Republic of China (35), representing the top five countries in terms of publication output. In terms of total link strength, which indicates the level of international research collaboration, the leading countries were the USA (163), Canada (131), Italy (120), Spain (115), and Germany (99).

**Figure 4:**
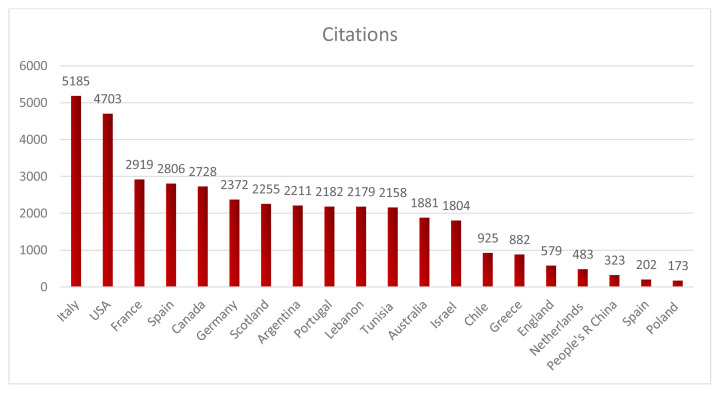
Number of citations by country in studies related to colchicine and cardiovascular research.

Italy ranked first in terms of total citation count (n = 5185), followed by the United States (4703), France (2919), Spain (2806), and Canada (2728). These countries constituted the top five in terms of citation impact within the literature on the cardiovascular effects of colchicine (Figure 4).

**Figure 5:**
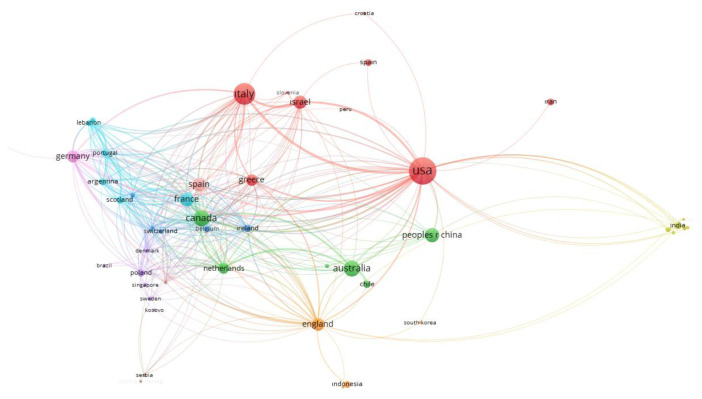
Network of international collaborations and citation networks among leading countries.

Figure 5 presents the international collaboration network among countries contributing to research on the cardiovascular effects of colchicine. In this network, each node represents a country, with node size proportional to the number of publications, and line thickness reflecting the strength of coauthorship links. The United States, Italy, Canada, and China appeared as the most central and highly connected countries, indicating a high level of collaborative activity. European countries such as France, Germany, Spain, and the Netherlands were also densely interconnected, forming a prominent regional cluster. Additionally, countries including India, Iran, South Korea, and Chile were positioned more peripherally, with fewer but still visible collaborative links.

Research on the effects of colchicine on the cardiovascular system has been predominantly driven by leading institutions worldwide. Maria Vittoria Hospital in Italy emerges as the leading contributor, with a total of 25 publications. McMaster University and the University of Western Australia followed with 16 publications each, while the University of Montpellier had 15. In terms of citation impact, the University of Montpellier ranked highest with 2428 citations, followed by the University of Glasgow (2180) and the Montreal Heart Institute (2113), despite their lower publication counts. Regarding collaboration, the University of Western Australia (57) and McMaster University (55) had the highest total link strengths, indicating strong international coauthorship networks. (Table 1)

Among a total of 2070 authors identified in the dataset, Massimo Imazio ranked first in terms of publication output, with 38 publications and 2538 citations. Other leading contributors included Adler Yehuda (26 publications, 1725 citations), Brucato Antonio (24, 1727), and Francois Roubille (19, 2363), indicating both productivity and high impact. Jean-Claude Tardif and Francois Roubille also had significant citation counts despite fewer publications (Table 2).

**Figure 6:**
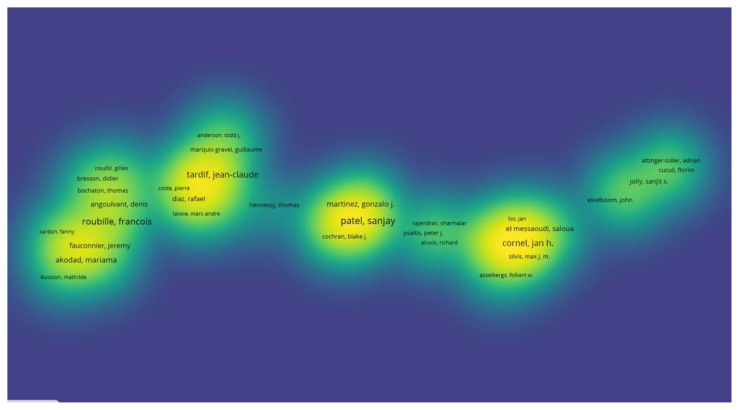
Heatmap of the top 100 most cited authors.

The author collaboration network, visualized in the heatmap, showed distinct clusters of researchers frequently coauthoring publications. Jean-Claude Tardif, Francois Roubille, Sanjay Patel, and Cornel Jan Hein appeared as central nodes in their respective clusters, indicating their strong presence in author networks within colchicine-related cardiovascular research (Figure 6).

Out of the 425 publications analyzed, 152 had not received any citations at the time of analysis. Among the 273 publications that had been cited at least once, the five most cited studies are listed in Table 3, with citation counts ranging from 448 to 1889 [1,5,7–9].

**Figure 7:**
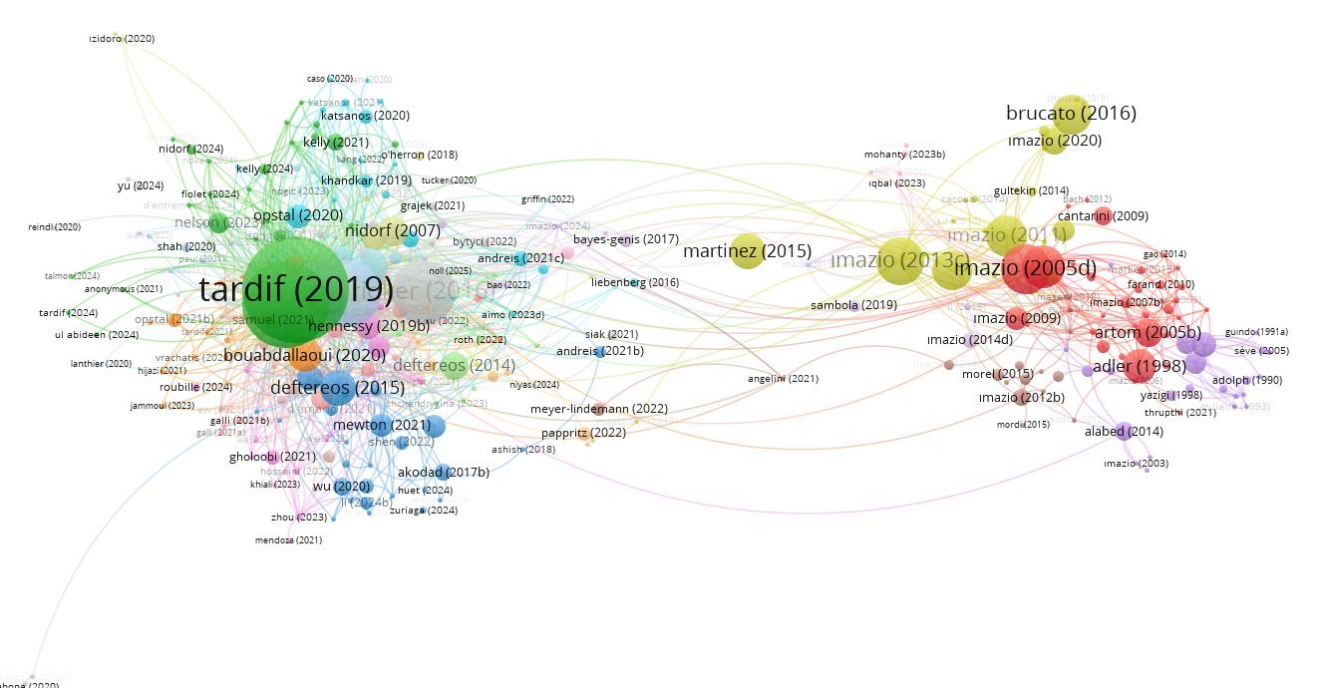
Citation network of the 100 most frequently cited publications.

Figure 7 presents the citation network of the most influential publications related to the cardiovascular effects of colchicine. In this network, highly cited publications such as Tardif et al. [7], Nidorf et al. [8], and Ridker [5] appear as central studies due to their strong citation connections. The network also shows clusters formed around specific authors, including Imazio, Brucato, and Tardif, which reflect areas of concentrated research activity and frequent scholarly contributions.

Table 4 presents the distribution of publications across major journals focusing on the cardiovascular effects of colchicine. The European Heart Journal published the highest number of articles (44), followed by Circulation (31) and the Journal of the American College of Cardiology (24). In terms of citation impact, Circulation led with 1458 citations and the strongest total link strength (336). The New England Journal of Medicine, despite contributing only 8 publications, received the highest number of citations (3549), indicating the exceptional influence of selected articles published in this outlet.

**Figure 8:**
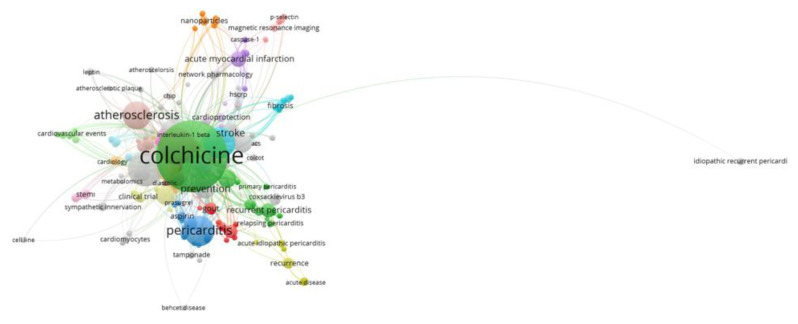
Most frequently used key word cooccurrence network.

A total of 978 keywords were used across the analyzed publications, with 363 keywords appearing more than once. The most frequently used keyword was “colchicine”, occurring 170 times, with the highest total link strength (631). Other frequently used terms included “inflammation” (68 times), “coronary artery disease” (52), “myocardial infarction” (45), and “pericarditis” (33). These keywords also demonstrated high link strength, particularly “coronary artery disease” (174), “myocardial infarction” (166), and “inflammation” (131), indicating their central role in the research network (Figure 8).

Table 5 displays the leading funding sources for studies investigating the cardiovascular effects of colchicine. The National Natural Science Foundation of China (NSFC) supported the highest number of studies (12 publications, 211 citations), followed by the National Health Medical Research Council (NHMRC) of Australia with 10 publications and 281 citations, and the Netherlands Organization for Health Research and Development with 8 publications and 289 citations. The Canadian Institutes of Health Research (CIHR) and the European Union (EU) each supported 5 studies, although with differing citation impacts (275 and 41 citations, respectively). Among private sector contributors, AstraZeneca supported 4 publications (146 citations), while Sanofi and Pfizer supported fewer studies (3 and 2 publications), with Sanofi appearing twice in the dataset.

## 4. Discussion

This bibliometric analysis provides a comprehensive overview of the research field on the effects of colchicine on the cardiovascular system, highlighting the main trends, influential publications, and key contributors in the field. The analysis reveals a growing interest in colchicine, with a sharp increase in publications, particularly after 2015. This trend correlates with the publication of pivotal clinical studies, such as LoDoCo and COLCOT, which have significantly influenced subsequent research in this area [6,7].

The earliest experimental evidence on the cardiovascular effects of colchicine dates back to the late 1970s. In a 1976 swine model study, Lee et al. investigated the impact of colchicine and methyl prednisolone on the development of aortic atherosclerosis. Their findings showed that colchicine did not prevent the progression of early or advanced lesions and had no effect on serum cholesterol levels, although it slightly worsened lesion formation in some cases. In contrast, methyl prednisolone was associated with increased lesion severity and elevated cholesterol levels. These results suggested a limited role for colchicine in atherosclerosis at the time [10]. A few years later, in 1981, Attie et al. explored the effect of colchicine on receptor-independent pathways of LDL degradation using WHHL rabbits. They reported that colchicine inhibited LDL catabolism in normal hepatocytes, but this effect was absent in mutant cells, pointing to a selective metabolic influence [11]. In 1983, Nakao and colleagues demonstrated that colchicine disrupts microtubule organization and inhibits smooth muscle cell migration, which is considered a key mechanism in the formation of atherosclerotic plaques. This study, cited over 100 times, proposed a plausible pathway through which colchicine could contribute to plaque reduction [12]. The first human study in this field was conducted by Lagrue et al. in 1985, involving 51 hypertensive patients with multiple vascular risk factors. Although colchicine did not alter lipid levels or blood pressure, it significantly improved microcirculatory dynamics, including arterial elasticity [13]. Collectively, these early findings laid the groundwork for the hypothesis that colchicine, through its antiinflammatory and cytoskeletal effects, may have therapeutic relevance in cardiovascular disease. This hypothesis has been supported by more recent large-scale clinical trials. By 2015, the mechanisms of colchicine were better defined, leading to broader clinical applications. Leung et al. demonstrated that its disruption of microtubule organization effectively suppresses inflammatory pathways, supporting its use in conditions such as osteoarthritis, pericarditis, and atherosclerosis [14].

In recent years, an increasing number of randomized controlled trials (RCTs) have been conducted to investigate the role of colchicine in cardiovascular disease management. For instance, Psaltis et al. assessed colchicine’s impact on coronary plaque morphology after myocardial infarction using optical coherence tomography. While colchicine had no significant effect on plaque characteristics in nonculprit segments overall, longer treatment duration was associated with increased fibrous cap thickness and reduced cap rupture, suggesting potential benefits for plaque stabilization over time [15].

Among the top five cited studies, the most cited study to date is the COLCOT trial, which has received 1889 citations, making it the leading reference in colchicine-related cardiovascular research. This landmark trial demonstrated that low-dose colchicine significantly reduced the risk of major ischemic cardiovascular events in patients who had recently experienced myocardial infarction. These events included myocardial infarction, stroke, and urgent coronary revascularization. The study reported a 23% reduction in the primary composite endpoint in the colchicine group compared to placebo (hazard ratio [HR]: 0.77, 95% confidence interval [CI]: 0.61–0.96). Although an increased incidence of pneumonia was observed in the colchicine group, the overall cardiovascular benefits and event reduction were considered the key clinical outcomes [7].

The LoDoCo2 trial, conducted by Nidorf et al. [8], is one of the most highly cited studies in colchicine research, with over 1245 citations, largely due to its rigorous design, large sample size, and clinically impactful findings. This randomized, double-blind, placebo-controlled trial evaluated the efficacy of low-dose colchicine (0.5 mg/day) in 5522 patients with chronic coronary disease over a median follow-up of 28.6 months.

Colchicine significantly reduced the primary composite outcome—including cardiovascular death, myocardial infarction, ischemic stroke, and revascularization—by 31% (HR: 0.69; 95% CI: 0.57–0.83; p < 0.001). The key secondary endpoint was also significantly improved (HR: 0.72; p = 0.007). Although noncardiovascular mortality was slightly higher in the colchicine group (1.9% vs. 1.3%), this difference was not statistically significant. The findings strongly support the role of colchicine as an effective adjunct to standard secondary prevention in stable coronary artery disease [8]. These findings were built upon earlier evidence from the LoDoCo (2013) trial, which stands as the third most cited study with 748 citations [6]. As a prospective, randomized, open-label trial involving 532 patients, LoDoCo1 was the first to demonstrate that low-dose colchicine added to standard therapy significantly reduced the incidence of acute coronary syndromes, cardiac arrest, and noncardioembolic stroke by 67% (HR: 0.33; p < 0.001). While limited by its open-label design and smaller sample size, LoDoCo1 laid the foundation for subsequent trials, particularly LoDoCo2, by highlighting the antiinflammatory potential of colchicine in atherosclerosis management [6]. The fourth most cited publication, authored by Ridker, provided a pivotal framework for recognizing inflammation, particularly interleukin-1 beta (IL-1β) signaling, as a central driver of atherosclerosis. This upstream perspective laid the scientific foundation for antiinflammatory strategies in cardiovascular disease. By inhibiting NLRP3 inflammasome activation and subsequent IL-1β release, colchicine targets critical components of this inflammatory cascade. In this context, Ridker’s work indirectly reinforces the therapeutic rationale for using colchicine in atherosclerotic disease management [5]. The fifth most cited study, conducted by Imazio et al., evaluated the efficacy of colchicine in patients experiencing a first episode of acute pericarditis. In this prospective, randomized, open-label trial, 120 patients were assigned to receive conventional therapy with or without the addition of low-dose colchicine (0.5–1 mg/day) for three months. The addition of colchicine significantly reduced the recurrence rate of pericarditis (10.7% vs. 32.3%) and improved symptom resolution at 72 h (88.3% vs. 63.3%) compared to standard treatment alone. These findings highlighted colchicine’s beneficial role in reducing recurrence and improving early symptom control in acute pericarditis, thereby expanding its clinical utility beyond coronary artery disease [9].

The analysis of the most cited publications highlights the pivotal role of a small number of high-impact studies in shaping the field of colchicine-related cardiovascular research. Notably, the top two articles, published in the *New England Journal of Medicine* by Tardif et al. and Nidorf et al., received 1889 and 1245 citations, respectively, underscoring their influence on clinical practice and scientific discourse. Authors such as Nidorf, Eikelboom, and Imazio appear multiple times among the top five, indicating their recurring contribution to landmark studies. These results reflect the importance of not only publication volume but also the quality and visibility of research, as a few well-executed trials have had a disproportionate impact on the academic landscape.

The journal distribution highlights the dominant role of high-impact cardiovascular journals in disseminating colchicine-related research. The European Heart Journal, with 44 publications and 594 citations, had the highest volume, while Circulation, with 31 articles, stood out in terms of impact, accumulating 1458 citations and achieving the strongest link strength (336). Although it featured only 8 publications, the New England Journal of Medicine garnered 3549 citations, demonstrating its role in publishing pivotal trials. Similarly, the Journal of the American College of Cardiology, with 24 publications and 1041 citations, also held a prominent position. These figures indicate that while some journals contribute heavily to research volume, others, like *NEJM* and *Circulation*, amplify the scientific visibility of individual studies due to their broad readership, high impact factor, and selectivity.

The analysis of author productivity and citation impact reveals a core group of influential researchers driving colchicine-related cardiovascular studies. Massimo Imazio led in publication volume, suggesting sustained involvement in the field, while Francois Roubille and Jean-Claude Tardif stood out for their high citation counts, pointing to the influence and visibility of their work. The heatmap visualization further highlights the collaborative structure of the field. Authors such as Patel, Tardif, and Roubille were positioned at the center of dense coauthorship networks, reflecting strong integration into multiinstitutional and possibly multinational studies. In contrast, authors with peripheral positions may represent emerging contributors or more independent research trajectories. These patterns underscore the importance of collaboration in enhancing scientific impact and shaping the discourse around colchicine in cardiovascular medicine.

Massimo Imazio emerges as the most prolific contributor in colchicine-related cardiovascular research, with 38 publications and 2538 citations to date. He is widely recognized for his pioneering work in the management of pericardial diseases. Notably, Adler Yehuda, another leading figure in the field with 26 publications and 1725 citations, has coauthored 17 of these studies with Imazio. Their collaboration has produced some of the most influential literature in the area, most prominently the COPE trial (Colchicine for Acute Pericarditis)—a prospective, randomized, open-label study that demonstrated the significant efficacy of colchicine in reducing symptom persistence and recurrence in patients experiencing a first episode of acute pericarditis. Imazio’s cardiovascular studies have also demonstrated colchicine’s role in secondary prevention following acute coronary syndrome, the prevention of postpericardiotomy syndrome, and restenosis after coronary angioplasty. In addition, his research on atrial fibrillation and heart failure has highlighted colchicine’s broader therapeutic potential in cardiovascular medicine. As such, Imazio not only leads in publication volume but, alongside key collaborators like Adler, plays a central role in shaping clinical perspectives and expanding the therapeutic scope of colchicine in cardiovascular care [9,16,17].

François Roubille and Jean-Claude Tardif rank among the most influential and productive researchers in the field of colchicine and cardiovascular disease, with 19 and 13 publications, respectively. Their collaborative work has played a pivotal role in shaping the clinical understanding of colchicine’s therapeutic role, particularly through their coauthorship of the landmark COLCOT trial, which demonstrated that low-dose colchicine significantly reduced major ischemic cardiovascular events following myocardial infarction. Building upon this foundation, Roubille and Tardif continued their collaboration in a 2024 prespecified analysis of the COLCOT trial, focusing on a high-risk subgroup of patients with type 2 diabetes. This study revealed a 35% relative reduction in the composite cardiovascular endpoint among diabetic patients treated with colchicine (HR: 0.65; 95% CI: 0.44–0.96; p = 0.03), along with a significantly lower incidence of recurrent events. These findings underscored the therapeutic relevance of colchicine beyond the general population, supporting its role in personalized cardiovascular risk reduction and further strengthening the scientific impact of both authors in the field [7–18]. Jean-Claude Tardif has also contributed to several related studies, including those examining the early initiation of colchicine (time-to-treatment initiation), its cost-effectiveness (analyses indicate that the addition of colchicine to standard-of-care therapy after MI is economically dominant and generates cost savings), pharmacogenomics, and its use in patients treated with percutaneous coronary intervention (PCI) [19–21].

Cornel Jan Hein, Peter L. Thompson, John W. Eikelboom, and Stefan M. Nidorf are among the most influential contributors in colchicine-related cardiovascular research, frequently coauthoring in several studies. Among these, Nidorf and Eikelboom have played central roles in the landmark LoDoCo1 and LoDoCo2 trials, which significantly advanced the understanding of colchicine’s role in chronic coronary artery disease and have received substantial citations. Their scientific contributions extend beyond randomized controlled trials. They have examined the long-term safety of colchicine, including its effects on hepatic and muscular enzymes, and its outcomes in patients with diabetes. These collaborative efforts have provided a broader clinical perspective, highlighting both the efficacy and safety profile of colchicine in secondary cardiovascular prevention. As a result, Nidorf and Eikelboom stand out not only in terms of publication activity but also total citations, underscoring the foundational nature of their research in this domain [6,8,22].

Sanjay Patel is a prominent contributor to colchicine research in the context of cardiovascular inflammation, with 13 publications and 1058 total citations. Patel’s contributions have been instrumental in elucidating the role of colchicine in modulating innate immune responses. His research has focused on the inhibition of the NLRP3 inflammasome, a key intracellular complex involved in the activation of interleukin-1β (IL-1β) and IL-18, which play a pivotal role in atherogenesis and plaque destabilization [23]. In another study conducted by Patel et al.,demonstrated that short-term colchicine therapy significantly attenuated the expression of IL-1β, IL-18, and IL-6 in patients with acute coronary syndromes, supporting its mechanistic role in suppressing vascular inflammation. The clinical implications of these findings were further supported by reductions in hsCRP levels and improvements in plaque stability markers in subsequent investigations. His contributions are visually underscored in the heatmap analysis, where Patel appears as a central node within a tightly clustered co-authorship network, reflecting strong collaborative engagement and academic influence in this domain. Collectively, his work has significantly shaped the current understanding of colchicine’s anti-inflammatory potential in cardiovascular therapy [24].

This bibliometric analysis highlights the global distribution and collaborative dynamics of colchicine-related cardiovascular research. As shown in Figure 3, the United States led in total publication output (128), followed by Italy (79), Australia, Canada, and China. These countries also demonstrated high total link strength values—particularly the USA (163), Canada (131), and Italy (120)—indicating their central roles in international research collaborations. When citation impact was considered (Figure 4), Italy emerged as the most influential country with 5185 citations, followed by the USA (4703), France (2919), Spain (2806), and Canada (2728). Italy’s strong citation performance may be attributed to the high-impact clinical trials conducted by authors such as Massimo Imazio and François Roubille, whose work on studies like COPE and COLCOT has significantly shaped the field.

Figure 5 further illustrates the global collaboration network. The USA, Italy, Canada, and China appeared as the most interconnected nodes, both in terms of publication volume and coauthorship strength. A prominent European cluster—comprising France, Germany, Spain, and the Netherlands—suggests dense regional collaboration. In contrast, countries like India, Iran, Chile, and South Korea occupied more peripheral positions, participating in fewer but still notable collaborations.

Regarding academic institutions, the findings of this study reveal that publication output and citation impact do not always align proportionally. While Maria Vittoria Hospital led in the number of publications (25), its prominence may be attributed to the presence of highly cited researchers who contributed to numerous influential studies, particularly on the role of colchicine in pericarditis [9,25,26]. Conversely, institutions such as the University of Montpellier, University of Glasgow, and Montreal Heart Institute demonstrated high citation counts despite producing fewer publications. This influence is likely driven by their coauthorship in the landmark COLCOT trial, which had a substantial impact on the field [7]. In terms of collaborative strength, the University of Western Australia and McMaster University stood out with high total link strength scores (57 and 55), suggesting a strong emphasis on international and interdisciplinary research partnerships. Such collaborations likely enhance the reach and visibility of their work within the global scientific community. These observations highlight the multifaceted nature of institutional impact, where both research quality and strategic collaboration play essential roles in shaping scholarly influence in colchicine-related cardiovascular research.

The funding analysis reflects a diverse landscape in which both public institutions and private sector contributors play critical roles in advancing colchicine-related cardiovascular research. Public agencies such as NSFC, NHMRC, and CIHR supported a relatively high volume of publications, many of which also received substantial citation counts, indicating a focus on broad scientific advancement. In contrast, private sector contributors like AstraZeneca, Sanofi, and Pfizer appear to concentrate their support on more targeted studies. These may be strategically aligned with their marketed products or therapeutic areas of interest, such as inflammation or cardiovascular risk. Their involvement likely reflects a commercial interest in expanding indications, supporting postmarketing evidence, or enhancing the clinical positioning of existing treatments.

## 5. Conclusion

This bibliometric analysis provides a comprehensive evaluation of the global research landscape regarding the cardiovascular effects of colchicine. The results demonstrate a clear increase in scholarly interest, particularly following pivotal clinical trials such as LoDoCo and COLCOT. A small group of highly influential authors and institutions have shaped the field through collaborative networks and high-impact publications. The United States and Italy emerged as global leaders in both productivity and scientific influence, supported by strong institutional and funding frameworks. Overall, colchicine has transitioned from a traditional antiinflammatory agent to a promising therapeutic option in cardiovascular disease prevention. Continued research, guided by clinical evidence and supported by international collaboration, will be essential in defining its long-term utility, safety, and cost-effectiveness in cardiovascular care.

Although some studies demonstrate limitations, the overall evidence supports the emerging role of colchicine in cardiovascular care. This comprehensive overview provides a valuable foundation for guiding future research and clinical practice in this evolving field.

## Figures and Tables

**Table 1:** Top 10 institutions producing research on the cardiovascular effects of colchicine.

Organization	Documents	Citations	Total link strength
Maria Vittoria Hospital	25	2013	42
Univ. Western Australia	16	493	57
McMaster University	16	309	55
University of Montpellier	15	2428	28
Chaim S. Medical Center	14	789	34
University of Sydney	13	1060	25
Tel Aviv University	13	892	27
Royal Prince Alfred H.	12	827	27
Montreal Heart Institute	7	2113	18
University of Glasgow	6	2180	21

**Table 2:** Overview of top authors by publications and citations in colchicine research.

Author(s)	Publications (n)	Total citations (n)
Massimo Imazio	38	2538
Adler Yehuda	26	1725
Brucato Antonio	24	1727
Francois Roubille	19	2363
Cornel Jan Hein	14	286
Stefan M Nidorf	14	1237
Jean-Claude Tardif	13	2023
Patel Sanjay	13	1058
John W Eikelboom	11	1470
Peter L Thompson	10	228

**Table 3:** Top 5 most cited publications.

Rank	The most influential publications	Citation (n)
1	Tardif et al. [7]	1889
2	Nidorf et al. [8]	1245
3	Nidorf et al. [6]	748
4	Ridker et al. [5]	669
5	Imazio et al. [9]	448

**Table 4:** Top 10 journals by number of documents and citations.

Source	Documents	Citations	Total link strength
European Heart Journal	44	594	164
Circulation	31	1458	336
Journal of the American College of Cardiology	24	1041	144
Journal of Cardiovascular Medicine	11	171	130
Frontiers in Cardiovascular Medicine	9	46	101
International Journal of Cardiology	9	143	45
New England Journal of Medicine	8	3549	301
Lancet	5	381	91
American Heart Journal	4	186	77
Journal of American Heart Association	4	343	23

**Table 5:** Funding sources for studies investigating the cardiovascular effects of colchicine.

Funding sources	Publications (n)	Total citations (n)
National Natural Science Foundation of China	12	211
National Health Medical Research Council of Australia	10	281
Netherlands Organization for Health Research and Development	8	289
Canadian Institutes of Health Research	5	275
European Union	5	41
German Research Foundation	5	122
AstraZeneca	4	146
Sanofi	3	115
Pfizer	2	33
Sanofi	2	132
